# Oral health locus of control in pregnant women with diabetes: a cross-sectional study

**DOI:** 10.1186/s12903-025-07401-4

**Published:** 2025-12-07

**Authors:** Zeynep Olcer, Busra Lekesiz Kapi

**Affiliations:** 1 Faculty of Health Science, Department of Nursing, Ankara Medipol University, Hacı Bayram Mahallesi Talatpasa Bulvari No:2, Altındag, Ankara, 06050 Turkey; 2 Department of Obstetrics and Gynecology, Medical Doctor, Ankara Etlik City Hospital, Varlık Mahallesi, Halil Sezai Erkut Caddesi Yenimahalle, Ankara, Turkey

**Keywords:** Dental, Diabetes, Gestational, Nursing, Oral health, Pregnancy

## Abstract

**Background:**

Perceptions of oral and dental health are crucial for health promotion. Pregnant women with diabetes are at increased risk for periodontal problems, making it important to understand their oral health locus of control to guide effective interventions. This study aimed to investigate the oral health locus of control among pregnant women with diabetes mellitus.

**Methods:**

This descriptive and cross-sectional study was conducted in the hospital’s perinatology clinics, involving 141 pregnant women with diabetes mellitus between July 2023 and 2024. The data were collected using the “Multidimensional Oral Health Locus of Control Scale”. For comparisons involving non-normally distributed data, the Mann-Whitney U test and the Kruskal-Wallis H test were used. In contrast, normally distributed data were analyzed using the Independent Samples t-test and One-Way Analysis of Variance. Approval from the ethics committee and institutional permission from the relevant hospital, and the participants’ informed consent were obtained.

**Results:**

Literate pregnant women had higher scores in the external locus of knowledge and chance locus of control, while those who planned their pregnancy had higher scores in the internal locus of control and external locus of practice. Statistically significant differences were found in sub-dimensions and total scores when oral health-related factors and pregnant women’s views on oral health, pregnancy, birth, and nutrition were examined.

**Conclusion:**

It is recommended that training and counselling should be provided to increase the perception of oral and dental health behaviours of pregnant women with diabetes mellitus, and factors such as women’s educational background and social environment should be taken into consideration during the training.

**Trial registration:**

not applicable.

**Supplementary Information:**

The online version contains supplementary material available at 10.1186/s12903-025-07401-4.

## Background

Pregnancy induces hormonal, immunological, and nutritional changes that increase susceptibility to oral and dental health problems, including periodontal disease [1–9]. Poor oral health in pregnancy has been linked to negative outcomes for both the mother and child, including gestational complications and early childhood caries [1–6, 10, 11]. Periodontal diseases are also linked to systemic conditions, including diabetes mellitus, and evidence suggests a bidirectional relationship between gestational diabetes mellitus (GDM) and periodontal health [12–15]. Disrupted oral health may impair maternal quality of life and contribute to suboptimal glycemic control in pregnant women with diabetes [3, 4, 11].

Individual perceptions of oral and dental health are critical in promoting and maintaining oral health [2, 5]. The concept of locus of control refers to a psychological measure that reflects individuals’ beliefs about whether their health is influenced by internal factors, such as their actions and decisions, or external factors beyond their control [15, 16]. In this context, an internal locus of control indicates the belief that outcomes depend on one’s own behaviors and efforts, while an external locus of control reflects the perception that outcomes are determined by chance, fate, or powerful others [17, 18]. Research indicates that patients with an internal locus of control exhibit better adaptation to diabetes management, whereas those with an external locus of control often experience more severe disease progression [15, 16].

Although locus of control has been widely studied in general diabetic populations, limited evidence exists regarding oral health locus of control among pregnant women with diabetes. Understanding how these women perceive control over their oral health is essential for guiding targeted interventions to support oral health maintenance, prevent complications, and promote a healthy pregnancy. Therefore, this descriptive study aimed to evaluate the perceptions of oral and dental health and the oral health locus of control among pregnant women with diabetes mellitus.

### Research questions

1. What are the oral and dental health habits of pregnant women with diabetes mellitus?
2. What is the oral health locus of control of pregnant women with diabetes mellitus?
3. Is there any difference between the oral and dental health habits of pregnant women with diabetes mellitus and the oral health locus of control?

## Methods

### Type, population, and sample of the study

This cross-sectional, descriptive study was carried out between July 17, 2023, and June 12, 2024. The study population comprised pregnant women who were diagnosed with diabetes after the necessary laboratory tests were performed by an internal medicine specialist and were therefore hospitalized in the perinatology clinic of the hospital. The sample size was determined using the formula for an infinite population [19]. It was calculated that there should be 139 pregnant women.

$$\mathrm n\:=\frac{t2\:\left(p.q\right)}{d2}$$

n: Number of individuals to be included in the sample. p: Frequency of occurrence of the case analyzed = 10% [20] (According to the relevant literature, the incidence rate of the event examined was calculated assuming that it was 10%).

q: Frequency of non-occurrence of the case analyzed = 90%.

t: The theoretical value found from the t table at a certain degrees of freedom and the identified error level = 1.96 (the theoretical t value at 𝛼 = 0.05 for ∞ degrees of freedom).

d: Standard error of the rate to be determined in the study = 0.05

$$\:\mathrm{n}=\frac{\left(1.96\right)2\:\left(0.1\mathrm{x}0.9\right)}{\left(0.05\right)2}\cong\:139$$

Three women who were unable to speak the native language, three women for whom a definite diagnosis cannot be established, six women who filled out an incomplete data collection form, and four women who declined to participate were excluded from the study. The study was conducted by a convenience sampling method, one of the non-probability sampling methods, and completed with 141 pregnant women with diabetes mellitus.

### Inclusion and exclusion criteria

Women who had diabetes, were aged 18 years and over, were voluntary to participate in the study, and were able to understand the statements in the information and scale form were included in the study. Women who had blood glucose monitoring at the time of the study but had not been diagnosed with definite diabetes were excluded.

### Variables of the study

The scores of the Multidimensional Oral Health Locus of Control Scale are the dependent variables of the study.

### Data collection tools

*Pregnant Woman Information Form*: This form was developed by the researchers based on a review of the relevant literature [11, 21–25] to collect descriptive data. It consists of 29 questions covering socio-demographic characteristics, oral and dental health habits, and related opinions. The form was created solely to obtain descriptive information, and it was not intended as a standardized measurement tool, therefore validity and reliability analyses were not conducted. An English version of the form is provided as a Supplementary File 1.

*“Multidimensional Oral Health Locus of Control Scale (MOHLCS)”*: It is a tool used to determine the health values of individuals and to assess how they perceive health-promoting behaviours and control power over health [23]. The questionnaire consisted of the MOHLCS, self-rated oral health (SROH), patterns of oral health behavior, and a socio-demographic section. This is a four-point Likert-type scale consisting of five subscales (Internal Locus of Control (belief in one’s own responsibility), External Locus of Knowledge (belief in dentists/experts), External Locus of Practice (belief in actions of others), Chance Locus of Control (belief in luck/fate), Locus of Socialization (influence of family, peers, culture)) and 26 items. None of the items on the scale is rated negatively. The items are rated as strongly disagree (1 point) and strongly agree (4 points). The total score of the scale ranges from 26 to 104 points. The scores of the subscale range from 11 to 44 on the internal locus of control subscale, 4 to 16 on the external locus of control subscale, 3 to 12 on the external locus of practice subscale, 6 to 24 on the chance locus of control subscale, and 2 to 8 on the locus of socialisation subscale [24]. These subscales reflect the extent to which individuals perceive their oral health outcomes as being determined by their own actions, by chance, by the dentist’s advice and recommendations, by the dentist’s preventive care, or by social influences such as family members, friends, and colleagues [25]. The validity and reliability study of the scale has been conducted, and Cronbach’s alpha coefficient was found to be 0.85 [24]. In this study, Cronbach’s alpha reliability coefficient was determined as 0.787.

### Data collection process

Firstly, the pregnant women were informed about the purpose and that their participation was voluntary, and the information obtained from the study would be used only for scientific purposes, and their information would be kept confidential. Afterwards, their verbal and written consents were obtained. Then, they were interviewed face-to-face in a suitable environment by ensuring privacy, and the researchers administered the questionnaire to most of the participants by reading the items one by one to them, and collected their responses in person.

### Ethical considerations

Approval from Ankara Medipol University Non-Interventional Clinical Trials Ethics Committee (IRB: E-81477236-604.01.01-2318, Date: 22/06/2023) and institutional permission from the Etlik City Hospital were obtained. In order to use the scale in the study, the necessary permissions were obtained from the authors. Informed consent of the participants was obtained. All steps of the study were carried out in accordance with the Declaration of Helsinki.

### Data analysis

Analysis of the data was carried out using SPSS version 25.0 (IBM Corporation, Armonk, NY, USA). Descriptive statistics included numbers, percentages, means, standard deviations, and range (minimum-maximum values), with the normality of data distribution verified with skewness and kurtosis values [26, 27]. For comparisons involving non-normally distributed data, the Mann-Whitney U test and the Kruskal-Wallis H test were used. In contrast, normally distributed data were analyzed using the Independent Samples t-test and One-Way Analysis of Variance. Post hoc analyses were executed using Bonferroni, Games-Howell, and Mann-Whitney U tests. The interpretation of results was based on a 95% confidence interval and a significance threshold of *p* < 0.05. Reliability was measured by Cronbach’s alpha coefficient [19].

## Results

The findings of the study indicated that 53.2% of the pregnant women with diabetes mellitus who participated in the study were aged between 27 and 35 years, and their mean age was 30.9 ± 6.0 (med. 30.0) (min-max. 18–47). All the women were married. Table 1 shows the socio-demographic characteristics of pregnant women with diabetes mellitus (Table 1).

**Table 1:** Socio-demographic, pregnancy, and general health condition characteristics of pregnant women with diabetes mellitus (*n* = 141)

Characteristics		*n*	%
Age	18–26	33	23.4
	27–35	75	53.2
	36 years and older	33	23.4
Employment	Employed	29	20.6
	Unemployed	112	79.4
Occupation	Unemployed/housewife	107	75.9
	Employed in the Health sector	6	4.3
	Employed in another sector	28	19.9
Educational background	Literate	11	7.8
	Primary school	32	22.7
	High school	50	35.5
	University and higher	48	34.0
Income status	Income is less than expenses	22	15.6
	Income equal to expenses	115	81.6
	Income is more than expenses	4	2.8
Gestational week	≤ 20 weeks	13	9.2
	≥ 21 weeks	128	90.8
Having a planned pregnancy	Yes	87	61.7
	No	54	38.3
Any health condition diagnosed by a physician	Diabetes mellitus	141	100.0
	Hypertension	17	12.1
	Hypothyroidism	17	12.1
	Other	21	14.9
Drug use	Yes	75	53.2
	No	66	46.8
A health problem diagnosed by a physician before pregnancy	Diabetes mellitus	26	18.4
	Other	16	11.3
	No	99	70.2
Smoking	Yes	15	10.6
	No	126	89.4
Alcohol consumption	Yes	3	2.1
	No	138	97.9

Comorbid diseases with diabetes are noted

When the obstetric and health-related conditions of the pregnant women were analysed, it was found that 90.8% of them were at gestational weeks of 21 or more, and 61.7% of them had planned their pregnancies (Table 1). The mean number of pregnancies, childbirths, miscarriages, abortions, and number of live children of the pregnant women were 2.5 ± 1.6, 1.1 ± 1.2, 0.3 ± 0.9, 0.0 ± 0.2, and 1.0 ± 1.1, respectively.

Table 2 shows the oral and dental health characteristics of pregnant women with diabetes mellitus. The dental health status of pregnant women at the time the data were collected was evaluated, and features such as caries, fillings, teeth pulled out but not filled, etc., were recorded, and it was determined that 40.4% of them had at least one carious tooth. When the distribution of MOHLCS subscale and total mean scores of the pregnant women was analysed, they had scores of 32.4 ± 5.3 (med. 32.0) (min-max. 17–44) on İnternal Locus of Control subscale, 8.6 ± 2.4 (med. 14.0) (min-max. 4–16) on External Locus of Knowledge subscale, 7.2 ± 2.0 (med. 14.0) (min-max. 3–12) on External Locus of Practice subscale, 10.4 ± 3.2 (med. 12.0) (min-max. 6–20) on Chance Locus of Control subscale, and 4.9 ± 1.6 (med. 5.0) (min-max. 2–8) on Locus of Socialisation subscale, and 63.8 ± 9.3 (med. 62.5) (min-max. 46–90) in overall MOHLCS.

**Table 2:** Characteristics of the diabetic pregnant women related to oral and dental health (*n* = 141)

Characteristics		*n*	%
Regular tooth brushing	No regular brushing	10	7.1
	1 time	44	31.2
	2 times	73	51.8
	3 times or more	14	9.9
Using dental floss in pregnancy	Yes	26	18.4
	No	115	81.6
Using a mouth rinse or mouth spray in pregnancy	Yes	36	25.5
	No	105	74.5
Attending regular dental check-ups (once a year)	Yes	26	18.4
	No	115	81.6
Time for the last dental check-up	I have never visited a dentist	11	7.8
	Within 1 year	40	28.4
	1 year ago	40	28.4
	2 years ago	21	14.9
	5 years or more	29	20.6
Are there any oral and dental health problems during pregnancy?	Yes	112	79.4
	No	29	20.6
Oral and dental health problems during pregnancy†	Toothache or caries	48	34.0
	Pain, tenderness, swelling, and bleeding in the gums	57	40.4
	Gum recession	15	10.6
	Dental sensitivity	33	23.4
	Bad odour and taste in the mouth	43	30.5
	Wound/candidosis in the mouth	7	5.0
	Mouth dryness	49	34.8
	Jaw joint pain	9	6.4
	Loose teeth	15	10.6
	Other	3	2.1
When the problem with the tooth appeared‡	During pregnancy	49	34.8
	Before pregnancy	49	34.8
	No memory	14	9.9
Has she visited a dentist about the problems she has suffered?	Yes	43	30.5
	No	98	69.5
Number of caries	None	84	59.6
	1	20	14.2
	2	18	12.8
	3 or more	19	7.1
Number of fillings	None	55	39.0
	1	16	11.3
	2	31	22.0
	3 or more	39	27.5
Number of teeth pulled out but not filled	None	71	50.4
	1	34	24.1
	2	16	11.3
	3 or more	20	14.1
Are pregnancy and periodontal health correlated?	Yes	78	55.3
	No	25	17.7
	I have no idea	38	27.0
Do periodontal diseases affect pregnancy?	Yes	48	34.0
	No	47	33.3
	I have no idea	46	32.6
Does oral and dental health during pregnancy affect childbirth?	Yes	13	9.2
	No	65	46.1
	I have no idea	63	44.7
Are nutrition and oral and dental health correlated during pregnancy?	Yes	81	57.4
	No	25	17.7
	I have no idea	35	24.8
Status of receiving training in oral and dental health before	Yes	10	7.1
	No	131	92.9
Assessment of oral and dental health§	Very Poor	2	1.4
	Poor	9	6.4
	Moderate	92	65.2
	Good	30	21.3
	Very Good	8	5.7

†Percentages of those who answered more than once are shown

‡Distribution of those who suffer from problems related to oral and dental health is shown

§Women assessed their own oral and dental health

When the socio-demographic and obstetric characteristics were compared with MOHLCS mean scores, no statistically significant difference was observed except for educational level (*p* > 0.05). Analysis of the mean scale scores according to educational level revealed a significant difference in the external locus of knowledge (F(3,137) = 4.737, *p* = 0.004) and chance locus of control (F(3,137) = 8.298, *p* < 0.001) subscales. To identify the groups between which the difference existed, Bonferroni and Games-Howell post-hoc analyses were done. Accordingly, the mean scores of illiterate pregnant women on the external locus of knowledge and chance locus of control subscales were higher than the scores of those who studied at a university. Analysis revealed that diabetic pregnant women on regular medication scored higher on the locus of socialisation subscale. In addition, women who had planned their pregnancies obtained significantly higher scores on both the internal and external locus of control subscales.

Table 3 shows the comparison of the subscales and the total MOHLCS score with some of the characteristics of oral and dental health habits and problems.

**Table 3:** Comparison of multidimensional oral health locus of control scale (MOHLCS) mean scores of the diabetic pregnant women with some of their characteristics about oral health (*n* = 141)

**Characteristics **		**MOHLCS** **Subscales and Total Score **											
		**Internal locus of control**		**External locus of knowledge**		**External locus of practice**		**Chance locus of control**		**Locus of Socialisation**		**Total Score**	
		**x̄±SD**	**Med.**	**x̄±SD**	**Med.**	**x̄±SD**	**Med.**	**x̄±SD**	**Med.**	**x̄±SD**	**Med.**	**x̄±SD**	**Med.**
Use of dental floss	Yes No	32.3**±**5.7 32.4**±**5.2	32.0 32.7	7.8±2.4 8.8±2.4	7.0 9.0	6.5±2.1 7.4±2.0	7.0 7.0	10.5±3.5 10.4±3.1	9.3 10.0	4.2**±**1.6 5.1**±**1.6	4.0 5.0	61.6**±**10.8 64.3**±**8.9	58.2 63.9
**Test statistic and p-value**		t=−0.104, p=0.917		t=−1.852, p=0.066		t=−1.997, p=0.048*		t=0.020, p=0.984		t=−2.263, p=0.025*		U= −2.104, p=0.035	
Use of mouth rinse	Yes No	33.1**±**4.6 32.1**±**5.5	32.5 32.0	7.6±2.1 9.0±2.4	7.5 9.0	6.8±2.4 7.4±1.9	7.0 7.0	9.4±3.0 10.8±3.2	8.0 10.5	4.5**±**1.7 5.1**±**1.6	4.0 5.0	61.6**±**9.6 64.6**±**9.1	60.5 63.0
**Test statistic and p-value**		t=0.970, p=0.334		t=−3.064, p=0.003*		t=−1.454, p=0.148		t=−2.303, p=0.023*		t=−1.910, p=0.058		U=−1.869, p=0.062	
Toothache/caries	Yes No	33.7**±**4.8 31.7**±**5.5	33.0 32.0	9.3±2.6 8.3±2.3	9.0 8.0	7.8±1.8 7.0±2.1	8.0 7.0	10.4±3.3 10.5±3.2	9.8 10.0	5.4**±**1.5 4.6**±**1.6	6.0 5.0	66.8**±**8.6 62.3**±**9.3	65.0 61.0
**Test statistic and p-value**		t=2.128, p=0.035*		t=2.240, p=0.027*		t=2.309, p=0.022*		t=−0.038, p=0.970		t=2.592, p=0.011*		t=2.805, p=0.006 *	
Bad odour/taste in the mouth	Yes No	33.2**±**4.4 32.0**±**5.7	33.0 32.0	9.3±2.6 8.4±2.3	9.0 8.0	7.7±2.0 7.1±2.0	8.0 7.0	10.6±2.8 10.4±3.4	10.0 9.3	5.4**±**1.7 4.7**±**1.6	6.0 5.0	66.4**±**9.3 62.7**±**9.1	65.0 62.0
**Test statistic and p-value**		t=1.208, p=0.229		t=2.124, p=0.035*		t=1.628, p=0.106		t=0.268, p=0.789		t=2.450, p=0.016*		t=2.159, p=0.033*	
Loose teeth	Yes No	30.8±4.2 32.6±5.4	31.0 32.8	8.2±2.4 8.7±2.4	8.0 8.5	6.6±1.1 7.3±2.1	7.0 7.0	9.4±2.4 10.6±3.2	9.0 10.0	5.0±1.4 4.9±1.7	5.0 5.0	60.1±6.3 64.3±9.5	62.1 62.7
**Test statistic and p-value**		t=−1.207, p=0.229		t=−0.686, p=0.494		t=−2.154, p=0.040*		t=−1.376, p=0.171		t=0.112, p=0.911		t=−1.643, p=0.103	
Pregnancy is correlated with periodontal health	Yes No No idea	33.5**±**4.8 30.6**±**6.8 31.4**±**4.9	33.0 31.0 32.0	8.2±2.5 9.2±2.0 9.1±2.3	8.0 9.0 9.0	7.1±2.2 7.4±1.8 7.5±1.8	7.0 8.0 7.0	9.4±2.8 11.8±3.2 11.7±3.3	8.3 12.0 12.0	4.9**±**1.7 5.5**±**1.6 4.5**±**1.5	5.0 6.0 4.5	63.3**±**9.1 64.6**±**11.5 64.4**±**8.2	62.0 61.0 64.4
**Test statistic and p-value**		F=3.718, p=0.027		F=2.624, p=0.076		F=0.411, p=0.664		**F=** 0.256, p=0.0001*		F=2.656, p=0.074		F=0.302, p=0.740	
Periodontal diseases affect pregnancy	Yes No No idea	33.2**±**4.3 32.7**±**5.5 31.2**±**5.9	32.0 32.0 33.0	8.3±2.5 8.5±2.2 9.2±2.6	8.0 8.0 10.0	7.1±2.2 7.0±1.9 7.6±1.9	7.0 7.0 7.3	9.6±2.8 10.3±2.9 11.5±3.6	9.0 9.7 11.5	5.0±1.7 4.8±1.7 4.9±1.5	5.0 5.0 5.0	63.5±8.3 62.0±9.6 66.1±9.6	63.0 61.0 65.5
**Test statistic and p-value**		F=1.853, p=0.161		F=1.891, p=0.155		F**=**0.977, p=0.379		F=4.734, p=0.010*		F=0.224, p=0.800		F=2.432, p=0.092	
Periodontal diseases affect childbirth	Yes No No idea	36.3±3.7 31.5±5.0 32.5±5.6	36.0 32.0 32.9	9.1±2.2 8.0±2.1 9.3±2.6	9.0 8.0 10.0	8.6±2.1 6.8±1.9 7.5±2.0	9.0 7.0 7.0	9.6±3.2 10.1±3.0 11.0±3.3	8.0 9.0 10.5	5.3±1.6 4.8±1.7 4.9±1.5	6.0 5.0 5.0	69.2±7.2 61.3±8.0 65.3±10.2	69.0 61.0 65.0
**Test statistic and p-value**		F=4.517, p=0.013*		F=5.021, p=0.008*		H=10.115, p=0.006*		F=1.839, p=0.163		F=0.508, p=0.603		F=5.610, p=0.005*	

F: One-way Anova Test, H: Kruskal-Wallis H Test, t: Independent Samples t Test, U: Mann-Whitney U Test

*p<0.05

A significant difference was observed in the internal locus of control (F(2,138) = 3.718, *p* = 0.027) and chance locus of control (F(2,138) = 10.256, *p* = 0.0001) subscales regarding the question “Is there any correlation between pregnancy and periodontal health?”. To determine between which groups these differences occurred, Bonferroni and Games-Howell post-hoc tests were performed. The analyses revealed that there was no significant difference between the groups in terms of internal locus of control, whereas the mean scores on the chance locus of control subscale were higher among women who believed that pregnancy and gingival health were not related and those who reported having no opinion on this issue (Table 3).

A statistically significant difference was found in the chance locus of control subscale for the question “Do periodontal diseases affect pregnancy?” (F(2,138) = 4.736, *p* = 0.010). Post-hoc analysis revealed that pregnant women who were uncertain about the effect of dental and gingival diseases on pregnancy had higher mean scores than those who believed such diseases could affect pregnancy (Table 3).

Analysis of the subscale and total scores for the question ‘Do periodontal diseases affect childbirth?’ revealed significant differences in the internal locus of control (F(2,138) = 4.517, *p* = 0.013), external locus of knowledge (F(2,138) = 6.050, *p* = 0.003), external locus of practice (H(2)/χ²(2) = 10.115, *p* = 0.006) subscales, and the total score (F(2,138) = 5.610, *p* = 0.005). To identify the groups between which the difference existed, the Bonferroni and Mann-Whitney U test were used for post-hoc analysis. Accordingly, the scores of the women who thought that periodontal diseases affected childbirth on the internal locus of control and external locus of practice subscales, and the overall MOHLCS, were higher than the scores of those who thought that they did not affect childbirth. The mean scores of those who stated that they had no idea, on the external locus of knowledge subscale, were higher than those who stated that periodontal diseases did not affect childbirth (Table 3).

When the opinions of the pregnant women about the correlation between nutrition and oral and dental health were evaluated, a significant difference was found in the subscales of external locus of control (F(2,138) = 6.430, *p* = 0.002) and chance locus of control (F(2,138) = 8.886, *p* = 0.0001). To identify the groups between which the difference existed, Bonferroni post-hoc analyses were done. Accordingly, the mean scores of the women, who stated that they had no idea about whether nutrition and oral and dental health were correlated, on the external locus of knowledge and chance locus of control subscales, and the mean scores of the pregnant women who thought that nutrition and oral and dental health were not correlated on the chance locus of control subscale were higher than those who thought that they were correlated.

## Discussion

The concept of locus of control reflects individuals’ beliefs about whether their health is influenced by internal factors, such as personal actions, or by external factors beyond their control [15, 16]. The perception of oral health control is an individual trait and affects the individual’s maintenance of health, utilisation of health services, and adaptation to preventive activities [2]. In this study, pregnant women with diabetes mellitus demonstrated moderate MOHLCS scores (63.88 ± 9.33), indicating a balance between perceived personal responsibility and external influences. It is recommended that healthcare professionals conduct more studies on this issue, and interventions should be made by taking into account the external dimension (social aspect) and internal dimension (perception, attitude, values, etc.) of women with chronic diseases such as diabetes.

While ensuring oral hygiene, the use of dental floss is as important as the frequency of brushing teeth [28]. Flossing may be affected by bleeding gums, but it is a habit that should be maintained [9]. Pregnant women are recommended to use dental floss once a day and to rinse their mouth every night with an alcohol-free mouth rinse [28, 29]. Oral hygiene practices, such as daily flossing and mouth rinse use, require time, skill, and resources. Women who did not engage in these behaviors scored higher on external locus of control dimensions, suggesting that perceived barriers and reliance on external factors may limit preventive behaviors. These findings are consistent with theoretical frameworks such as the Health Belief Model, where perceived susceptibility and severity of oral health problems influence motivation, and Self-Efficacy Theory, which posits that confidence in one’s ability to perform health behaviors is critical for engagement [30, 31]. Pregnancy is a unique period when women can benefit from oral and dental health services [29]. Educational programs targeting skill development, increasing awareness of personal responsibility, and addressing environmental and social barriers are therefore essential.

Individuals with an internal locus of control are more likely to engage in preventive health behaviors, whereas those with an external locus of control are more inclined to attribute health outcomes to external circumstances or powerful others [16]. In this study, women who reported toothache or caries problems demonstrated higher mean scores not only on the internal locus of control subscale but also on external locus of control, external locus of practice, locus of socialisation, and overall MOHLCS scores. These findings may be better understood within established theoretical frameworks of health behavior. According to the Health Belief Model, experiencing a health threat such as dental pain may heighten perceived susceptibility and severity, thereby motivating individuals to take action if they believe they have sufficient control over the outcome [30]. Similarly, Self-Efficacy Theory suggests that confronting a health problem may enhance individuals’ belief in their own ability to perform health-promoting behaviors, which can account for elevated internal locus of control scores [31]. At the same time, the increase in external locus of control dimensions indicates that these women may also rely on social and environmental factors when responding to oral health problems. This duality underscores the importance of integrating both individual responsibility and social support in oral health promotion strategies. Healthcare professionals, therefore, could leverage family involvement and peer groups in oral and dental health training programs to strengthen self-efficacy and reinforce positive behavioral change.

It has been reported that pregnant women often have insufficient knowledge about oral diseases [9]. Education about oral health has been recommended as part of prenatal care programs, thus women’s oral hygiene can be improved [8, 9]. Ebinghaus et al. [8] reported in their study that many women did not receive any information on oral health during pregnancy. In the study by Al Humaid et al. [5], it was determined that 74.4% of pregnant women did not receive any oral health education during pregnancy. In this study, only 7.1% of pregnant women with diabetes mellitus reported that they received information on oral and dental health previously. This rate indicates that healthcare professionals remain incapable in oral and dental health training. It is reported that oral health training and support should be a part of standard diabetes management [12]. Therefore, oral and dental health care should be incorporated into the care of pregnant women with diabetes.

Women’s educational level is one of the factors that contribute to an increased level of oral health knowledge [5]. Azab et al. [7] found that educated pregnant women were more likely to maintain good oral hygiene. Illiterate women had higher external locus of knowledge and chance locus of control scores compared to university graduates, likely reflecting lower health literacy and a tendency to attribute outcomes to chance or external influences [16]. Tailoring educational interventions to women’s educational backgrounds can enhance their understanding of oral health and empower them to adopt preventive behaviors.

The findings also indicate a substantial knowledge gap regarding the relationship between oral health, pregnancy, and childbirth. Women who were unaware of correlations between periodontal health and pregnancy or childbirth exhibited a higher chance and an external locus of control score. This suggests that a lack of information may lead women to perceive adverse outcomes as beyond their control. Considering that poor oral health during pregnancy is associated with negative maternal and fetal outcomes [3, 6, 11–13], healthcare professionals should integrate oral health education into prenatal care, particularly for women with chronic conditions such as diabetes. Moreover, it has been reported that individuals with diabetes have a low awareness of the mutual correlation between diabetes and periodontal disease [22]. Healthcare professionals should inform pregnant women, especially pregnant women with chronic diseases such as diabetes, about the effects of this disease on oral and dental health, pregnancy, childbirth, and the fetus. Incorporating such training into educational curricula and in-service training can strengthen the capacity of professionals to provide effective guidance.

### Limitations

The limitation of the study is that it is being conducted in a single centre and only in the perinatology clinics of the relevant hospital. This restricts the generalizability of the findings to a broader population of pregnant women with diabetes. This study is a survey study based on the declarations of the participants. This is another limitation of the study. The researchers administered the questionnaire to most of the participants by reading the items one by one to them, and collected their responses in person to avoid any problems that may arise due to this situation. Moreover, participation in the study was voluntary, and participant confidentiality was strictly maintained, thereby minimizing the likelihood of recall or response bias.

### Relevance for clinical practice

This study highlights the importance of integrating oral and dental health into the care of pregnant women with diabetes. The findings may support healthcare professionals in paying greater attention to oral and dental health when providing counselling and education during pregnancy. In addition, the study may help increase awareness among pregnant women regarding the significance of maintaining oral and dental health as part of overall maternal care.

## Conclusions

The oral health locus of control of pregnant women with diabetes mellitus was at a moderate level. Women’s oral health locus of control affected the use of oral and dental hygiene materials such as dental floss and mouth rinse. The pregnant women with diabetes mellitus had a low rate of attending regular dental check-ups and receiving training on oral and dental health. Toothache/caries, bad odour/taste in the mouth, and loose teeth were affected by the oral health locus of control. Based on these results, the following are recommended: Healthcare professionals should provide more training and counselling to pregnant women with diabetes mellitus about oral and dental health, considering the educational background of women in prenatal care. Campaigns should be organised to enhance self-efficacy and to raise the awareness of pregnant women that they are responsible for improving their oral and dental health. Oral health training and support should be made a standard part of diabetes management in pregnancy, and women should be encouraged to attend dental check-ups. Further studies, including pregnant women with and without chronic diseases, particularly non-diabetic pregnant women as a potential control group, are recommended to explore the differences in oral health perceptions and locus of control.

## Supplementary Information

Supplementary Material 1.

## Data Availability

All data generated or analyzed during this study are included in this article. Additional supporting data are available from the corresponding author (ZO) upon reasonable request.

## Abbreviations

GDM: Gestational Diabetes Mellitus
MOHLCS: Multidimensional Oral Health Locus of Control Scale
SROH: Self-Rated Oral Health
IRB: Institutional Review Board
