# Supplementary material for: Oral health locus of control in pregnant women with diabetes: a cross-sectional study
Source: BMC Oral Health. 2025 Dec 7;26:71. doi: 10.1186/s12903-025-07401-4 (PMC12797511; doi:10.1186/s12903-025-07401-4)
Supplement: Supplementary file 1 — Supplementary Material 1. [file 12903_2025_7401_MOESM1_ESM.pdf]

Pregnant Woman Information Form (English Version)

Questionnaire ID: .... **Date:** ....

1. **Age:** .... (years)
2. **Height:** ....
   Pre-pregnancy weight: ....
   Current weight: ....
   BMI: ....
3. **Education level:**
   1. Illiterate 2. Literate 3. Primary education
   4. Secondary education 5. Higher education
4. **Employment status:**
   1. Employed 2. Unemployed
5. **Do you have social security/health insurance?**
   1. Yes 2. No
6. **Current gestational week:** ....
7. **Obstetric history:**
   G: .... **P:** .... **A:** .... **D&C:** .... **Y:** ....
   (G: gravida, P: parity, A: abortion, D&C: dilation & curettage, Y: living children)
8. **Was this pregnancy planned?**
   1. Yes 2. No
9. **Do you have any medical conditions accompanying your pregnancy?**
   ....
10. **Do you use any regular medication?**
    1. Yes (Please specify: ....)
    2. No
11. **Did you have any medical condition before pregnancy?**
    1. Yes (Please specify: ....)
    2. No
12. **Do you smoke?**
    1. Yes (Number of cigarettes per day: ....)
    2. No
13. **Do you brush your teeth?**
    1. Yes (Number of times per day: ....)
    2. No
14. **Do you use dental floss and/or interdental (interproximal) brush?**
    1. Yes (Number of times per day: ....)
    2. No
15. **Do you use mouthwash and/or oral spray?**
    1. Yes 2. No
16. **Do you attend regular dental check-ups? (once a year)**
    1. Yes 2. No

17. When was your last dental visit?

1. I have never visited a dentist
2. 6 months ago
3. 1 year ago
4. 2 years ago
5. 5 years ago or more

18. Have you experienced any oral or dental health problems during your pregnancy? If yes, what were they? *(More than one option may be selected)*

1. Toothache or dental caries
2. Pain, sensitivity, swelling or bleeding in the gums
3. Gum recession
4. Dental sensitivity
5. Bad breath or taste in the mouth
6. Oral sores/thrush
7. Dry mouth
8. Temporomandibular joint pain
9. Tooth mobility
10. Other (please specify: ....)
11. No, I did not experience any oral or dental health problems during my pregnancy.

19. Please indicate when the dental/oral problem first occurred:

1. During pregnancy   2. Before pregnancy   3. No memory

20. Did you visit a dentist regarding these problems?

1. Yes
2. No (please specify the reason: ....)

21. Please indicate the number of decayed, filled, or extracted but not yet filled teeth:

Number of decayed teeth: ....

Number of filled teeth: ....

Number of extracted but unfilled teeth: ....

22. Do you believe that your pregnancy has affected your oral and dental health?

1. Yes   2. No   3. I have no opinion

23. Do you believe that your existing health condition during pregnancy affects your oral and dental health?

1. Yes   2. No   3. I have no opinion

24. In your opinion, is there a relationship between pregnancy and oral/dental health?

1. Yes   2. No   3. I have no opinion

25. Do you believe that oral and dental diseases affect pregnancy?

1. Yes   2. No   3. I have no opinion

26. Do you believe that oral and dental health during pregnancy affects childbirth?

1. Yes   2. No   3. I have no opinion

27. Is there a relationship between nutrition during pregnancy and oral/dental health problems?

1. Yes   2. No   3. I have no opinion

28. Have you received any education regarding oral and dental health?

1. Yes (please specify from whom: ....)
2. No

29. How would you rate your oral and dental health?

**1**	**2**	**3**	**4**	**5**
Very poor	Poor	Moderate	Good	Very good

Developed by the researchers based on the literature [11, 21-25].
